# Transcutaneous Electrical Cranial-Auricular Acupoint Stimulation vs. Escitalopram for Patients With Mild-to-Moderate Depression (TECAS): Study Design for a Randomized Controlled, Non-inferiority Trial

**DOI:** 10.3389/fpsyt.2022.829932

**Published:** 2022-05-10

**Authors:** Sichang Yang, Zongshi Qin, Xinjing Yang, Mei Yan Chan, Shuiyan Zhang, Peijing Rong, Xiaobing Hou, Guixing Jin, Fengquan Xu, Yong Liu, Zhang-Jin Zhang

**Affiliations:** ^1^Department of Chinese Medicine, The University of Hong Kong Shenzhen Hospital (HKU-SZH), Shenzhen, China; ^2^LKS Faculty of Medicine, School of Chinese Medicine, The University of Hong Kong, Pokfulam, Hong Kong SAR, China; ^3^Institute of Acupuncture and Moxibustion, China Academy of Chinese Medical Sciences (CACMS), Beijing, China; ^4^Beijing First Hospital of Integrated Chinese and Western Medicine, Beijing, China; ^5^Department of Mood Disorders, The First Hospital of Hebei Medical University, Shijiazhuang, China; ^6^Guang’anmen Hospital, China Academy of Chinese Medical Sciences, Beijing, China; ^7^Department of Radiology, The Affiliated Traditional Chinese Medicine Hospital of Southwest Medical University, Luzhou, China

**Keywords:** depression, transcutaneous electrical cranial-auricular acupoint stimulation, escitalopram, non-inferiority, randomized controlled trial

## Abstract

**Background:**

Previous studies in animals and humans indicated that transcutaneous vagus nerve stimulation (tVNS) and transcutaneous electrical acupoint stimulation (TEAS) on trigeminal nerve-innervated forehead acupoints can relief the symptoms of depression. However, due to the limited investigations on these two interventions, more research are needed to confirm their efficacy in depression. To improve the efficacy of the single treatment, we combined two treatments and created a novel non-invasive stimulation, transcutaneous electrical cranial-auricular acupoint stimulation (TECAS). To assess the efficacy and safety of TECAS, we compare it with a selective serotonin reuptake inhibitor (SSRI), escitalopram, for the treatment of depression.

**Methods/Design:**

This is a multi-center, non-inferiority, randomized controlled trial that will involve 470 patients with mild to moderate depression. Patients will be randomly assigned to either the TECAS group or the escitalopram group in a 1:1 ratio. The TEAS group will receive two sessions of treatments per day for 8 consecutive weeks, and the escitalopram group will receive 8 weeks of oral escitalopram tablets prescribed by clinical psychiatrists as appropriate for their condition. The primary outcome is the clinical response as determined by Montgomery-Åsberg Depression Rating Scale (MADRS) scores at week 8, with −10% as the non-inferior margin. The secondary outcomes include the response rate determined by 17-item Hamilton Depression Rating Scale (HAMD-17), remission rate, changes from baseline in the scores on the MADRS, the HAMD-17, the Hamilton Anxiety Rating Scale (HAMA), the Pittsburgh Sleep Quality Index (PSQI), and the Short Form 36 Health Survey (SF-36).

**Discussion:**

This will be the first randomized controlled trial to compare the efficacy of TECAS with escitalopram for depression. If effective, this novel intervention could have significant clinical and research implications for patients with depression.

**Clinical Trial Registration:**

[ClinicalTrials.gov], identifier [NCT03909217].

## Introduction

Major depressive disorder (MDD) is highly prevalent worldwide, representing a significant global burden (1). Currently, second generation agents including antidepressants such as selective serotonin reuptake inhibitors (SSRIs) and serotonin and norepinephrine reuptake inhibitors (SNRIs) are commonly recommended as a first-line treatment option for depression (2–4). However, attributed to the neurological and gastrointestinal adverse effects, the adherence to antidepressants were low, with nearly half no longer refilling their first prescription (5).

Brain stimulation techniques are increasingly used for treatment-resistant depression (6). Among them, vagus nerve stimulation (VNS) uses electrical stimulation to provides chronic bilateral activation of brain circuits (7). Long-term VNS has been demonstrated to be correlated to sustainable patient benefits (8–11). In 2005, the US FDA has approved VNS for managing treatment-resistant depression. To date, more than 100,000 patients with psychiatric or neurological indications are treated with VNS each year (12). Conventional implantable VNS (iVNS) requires a surgical implantation procedure, which restricts its accessibility and increases the financial costs and complications, such as voice changes, bradycardia, dyspnea, cough and pain (2, 13). To alleviate these barriers arising from the invasive process and to improve the tolerability and practicability of VNS, transcutaneous VNS (tVNS) has been developed. Different from iVNS modalities, transcutaneous VNS (tVNS) uses electrodes placed on the skin surface to produce electrical stimulation to the auricular branch or cervical branch of the vagus nerve. The available evidence suggests tVNS’s effects in alleviating depressive symptoms (14, 15). However, depression guidelines and systematic reviews consider VNS more as an adjunct therapeutic method to existing primary treatments (2, 3, 8, 13). The effectiveness of its short-term treatment remains controversial (16–18). A randomized controlled trial with 235 treatment-resistant depression patients failed to show a significant advantage of short-term VNS therapy over placebo (17). Therefore, we expect to involve additional neurostimulation to enhance the effectiveness of VNS in the treatment of depression.

Previous studies in animals and humans and our pilot study (unpublished data) have shown that electrical acupuncture in the cranial region can improve symptoms of depression (19–26). Similarly, the Evidence-based Guidelines of Clinical Practice with Acupuncture and Moxibustion–Depression (Revised) recommends the cranial acupoints Yin-Tang (EX-HN3) and Bai-Hui (DU20) for depression (27). Those forehead acupoints are found innervated by the trigeminal nerve (28), while the stimulation process activates the sensory pathways of the trigeminal nerve (29). However, as an invasive treatment, electro-acupuncture is prone to clinical side effects such as infection, needling pain, and subcutaneous hematoma. In addition, an experienced acupuncturist is required to perform the treatment. All these reasons reduced adherence as well as cost-effectiveness of acupuncture treatment (30). Therefore, a novel method for acupoints stimulation called transcutaneous electrical acupoint stimulation (TEAS) was developed. It is easy-to-use and non-invasive, which increases safety by avoiding the process of needling and the resulting side effects of pain and needle phobia (31). Although more clinical evidence on cranial TEAS for depression is still needed, results of a 8-week open pilot study including 11 patients with moderate to severe depression and taking concurrent antidepressant medication suggested that nightly transcutaneous trigeminal nerve stimulation (V1 branch) can significantly improve the depressive symptoms assessed by clinician-rated or self-rated scales (32). Moreover, Shiozawa’s team conducted a phase II, randomized, sham-controlled trial of 40 patients with MDD to test the effects of a 10-day stimulation of the supraorbital branches of the trigeminal nerve while taking concurrent antidepressant medication. The results showed that although there was a significant interaction for changes in depressive symptoms over treatment and control groups across three assessments; *post-hoc* analyses found significant differences between depressive symptoms at baseline and after intervention protocol, as well as between at baseline and during 1-month follow-up (33). These evidence suggests the possible efficacy of using transcutaneous acupoint trigeminal nerve stimulation for depression. As TEAS and tVNS are both safe and practical methods producing stimulation with similar principles and equipment requirements, combining the two does not place an additional burden on patients. Therefore, we combined transcutaneous electrical stimulation of the Yin-tang and Bai-hui acupoints with tVNS to create a novel electrical stimulation method called transcutaneous electrical cranial-auricular acupoint stimulation (TECAS). In addition to the stimulation of the vagus nerve provided by tVNS, this new method involves TEAS stimulation of the trigeminal nerve through the relevant acupoints. We expect that the addition of this transcranial trigeminal nerve stimulation could be additive to the effectiveness of the original auricular vagus nerve stimulation for depression.

To evaluate the efficacy and safety of TECAS in improving depressive symptoms, accompanying anxiety, insomnia, and the quality of life for patients with mild-to-moderate depression, we designed a randomized controlled trial with non-inferiority design comparing the efficacy of TECAS and a commonly used, representative SSRI, escitalopram. The results of this study will provide robust evidence on whether TECAS has the potential to become a safe and effective treatment for depression.

## Methods and Analysis

### Design

**FIGURE 1:**
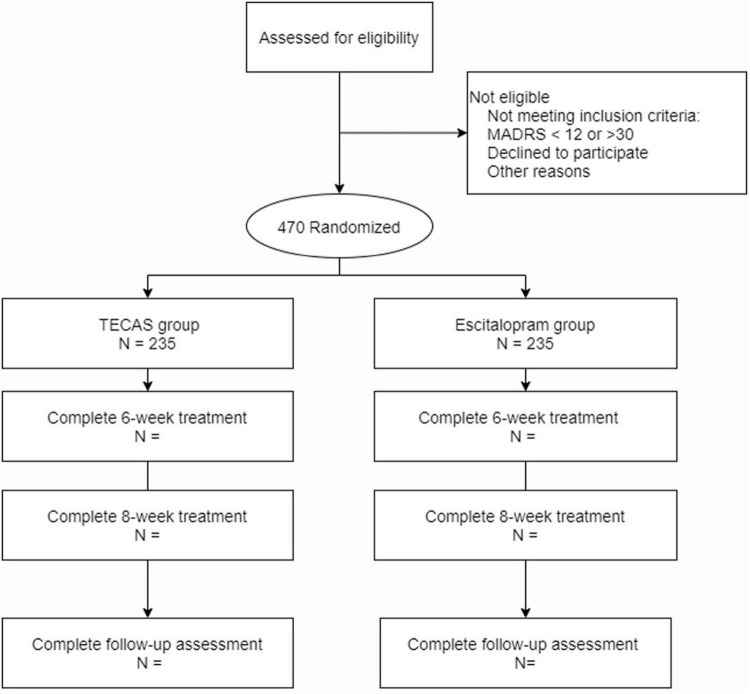
Study flow chart.

The TECAS trial is a multi-center, non-inferiority, randomized controlled trial and will be conducted simultaneously at five hospitals in China including The University of Hong Kong-Shenzhen Hospital; Guang’anmen Hospital of China Academy of Chinese Medical Sciences; Beijing First Hospital of integrated Chinese and Western Medicine; The First Hospital of Hebei Medical University; and Southwest Medical University, Hospital of Traditional Chinese Medicine. This study protocol is developed according to the Standard Protocol Items: Recommendations for Intervention Trials (SPIRIT) checklist (34) and has been approved by the Research Ethical Committee of The University of Hong Kong Shenzhen Hospital (HKU-SZH) (project approval number: 2019044; approved on 28 February, 2019), and all participating medical sites. The study protocol has been previously registered at ClinicalTrials.gov (NCT03909217). Figure 1 presents a study flow chart of the progress of patients through the trial.

### Participants

Four hundred and seventy patients with mild-to-moderate depression will be recruited *via* hospitals, posters, and web advertisements. Participants will be recruited after referral to specialty psychiatrists at five hospitals. We will recruit adults aged 18–70 years who have a confirmed diagnosis based on the International Classification of Diseases 10th Edition (ICD-10, F32) of mild-to-moderate depression. A patient will be eligible if their current episode shows a Montgomery-Åsberg Depression Rating Scale (MADRS) score of at least 12 and less than 30; and all responses to the Suicide Severity Scale (C-SSRS) were “no” or scored <3 points. The exclusion criteria including the patients with severe diseases of heart, brain, liver, kidney or hematopoietic system, with acute diseases, infectious diseases or malignant tumors; with any history of psychosis or mania; with cognitive disorders or personality disorders; with serious suicidal ideation or behaviors; the patients who unable to stop taking relevant drugs according to the treatment requirements, or pregnant. Professional psychiatrists will be responsible for the assessment of the mentioned disorders in exclusion criteria based on the appropriate clinical diagnostic criteria in China. Participants were prohibited to use any other drug or non-pharmacological therapy, including phytomedicine, antidepressants, and physical therapy methods or ways, which may affect the depression symptoms during the treatment period. All participants have to provide written, informed consent.

### Randomization

Patients will be randomly allocated (1:1) to groups receiving either escitalopram or TECAS. Stratified blocked randomization codes will be generated by PROC PLAN using SAS software, which will be stratified by the study site. The research assistants will use the randomization codes to produce opaque, sealed envelopes. Each envelope will be labeled with a participant-specific randomization identification number and contain a treatment allocation number. After the collection of patients’ basic information, participants will be assigned a randomization identification number by study staff. By using a central randomization system composed of free telephone, short message, and E-mail, the randomization identification number and treatment allocation will be accessed after participants received their baseline clinical evaluation. Participants, clinical psychiatrists, and treatment technicians will be aware of the treatment condition. The staff assessing treatment outcomes will be blinded to treatment condition because they will be segregated in a different clinic area. The participants’ treatment allocation will be required not to be discussed with these staff or other participants.

### Intervention Procedure

**FIGURE 2:**
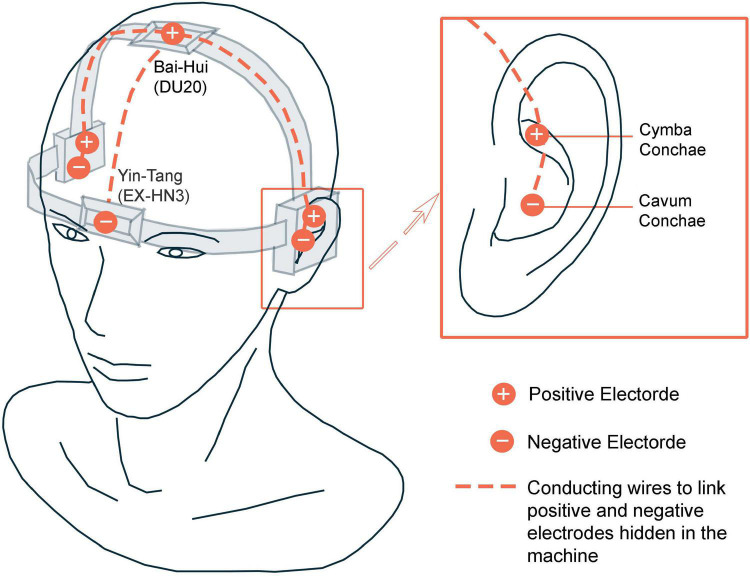
Illustration of Transcutaneous electrical cranial-auricular acupoint stimulation (TECAS).

Before receiving interventions, participants will receive related detection including blood routine, urine routine and electrocardiogram, or to provide the results of a physical examination within 2 weeks. The TECAS treatment consisted of two sessions per day for 8 consecutive weeks. For patients who will receive TECAS treatment, cranial Yin-Tang (EX-HN3), Bai-Hui (GV20) acupoints electrical stimulation and auricular vagus nerve electrical stimulation will be used. The patients will take a supine position or sitting position and will use the SDZ-IIB (Hwato, Jiangsu) electronic acupuncture instrument equipped with two pairs of cranial and auricular electrodes. The cranial electrodes will be located in EX-HN3 and GV20 acupoints, and the auricular electrodes will be located in the bilateral distribution area of the vagus nerve of the cavity and cymba of the auricular concha (Figure 2). The cranial and auricular electrodes stimulation will last for 30 min each session with a disperse-dense wave. This duration has been applied in several previous related electrical stimulation experiments, whose results suggest its efficacy and safety (19, 33, 35). Previous study on electroacupuncture indicated that low frequency could produce broader neuromodulation compared to higher frequency (36). Also, due to safety considerations, vagus nerve stimulation is conventionally constrained to administration at fairly low frequencies (≤30 Hz) (37). Considering the above literature and our clinical experience, the frequency will be adjusted to 4/20 Hz (4 Hz for 5 s, 20 Hz for 10 s, alternately). The intensity will be adjusted to be tolerable without pain. Patients will be treated at home twice a day as required, once in the morning and once in the evening (the evening treatment could be done 30 min before bedtime). Prior to the start of home treatment, the study team will hold a training session for participants to ensure that they are familiar with the precautions for using the device.

For patients who will receive escitalopram treatment, oral administration escitalopram tablets will be prescribed by clinical psychiatrists with respect to patients’ conditions for 8 consecutive weeks. The usual dosage is 5 mg daily in the first week and 10 mg daily from the second week. Clinicians can increase the maximum daily dose to 20 mg daily depending on the patient’s response, condition, and course of the disease. For elderly patients (>65 years of age), it is recommended to start the treatment at half of the usual starting dose described above, and the maximum dose should be reduced accordingly.

In terms of the drug combination, for patients with severe insomnia, the treatment group and control group can be combined with weak diazepam or non-benzodiazepine sedative drugs; the drug use should be recorded in the case report form (CRF). The MADRS score, the 17-item Hamilton Depression Rating Scale (HAMD-17) score, the Hamilton Anxiety Rating Scale (HAMA) score, the Pittsburgh Sleep Quality Index (PSQI) score and the Short Form 36 Health Survey (SF-36) will be assessed by trained research assistants at baseline, 2, 4, 8, and 12 weeks. Adverse events (AE) of all participants will be assessed and evaluated during the whole procedure, as well as laboratory tests (blood routine, urine routine, and electrocardiogram) if needed.

### Outcomes

#### Primary Outcome

The primary outcome is clinical response at the end of treatment. The responder is defined as a ≥50% reduction from baseline MADRS at week 8. MADRS is a validated, self-reported and well-recognized questionnaire for depression (38–40). It consists of nine items, with the total range of 0–60 (higher scores indicate more severe condition). Previous research estimated the minimal clinically important difference (MCID) of MADRS to be 1.6–1.9 points changed from baseline (41).

#### Secondary Outcomes

**FIGURE 3:**
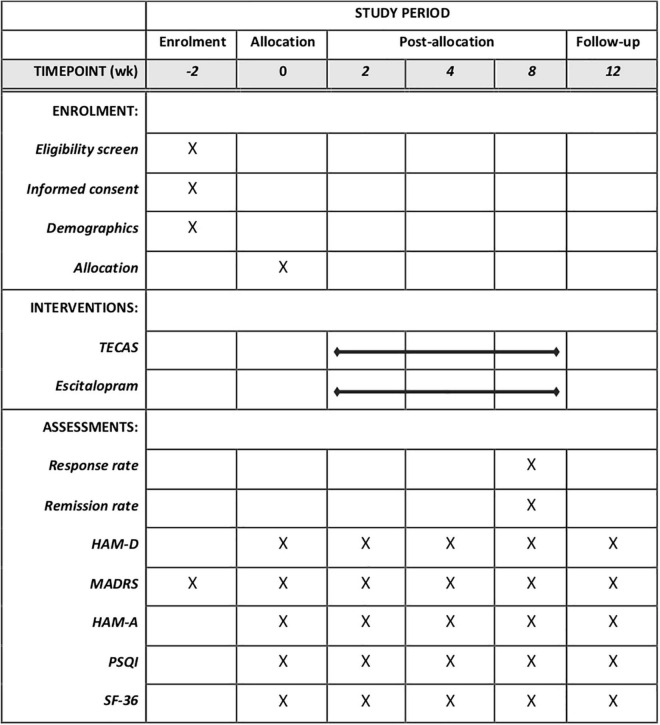
Standard protocol items: recommendations for intervention trials (SPIRIT) schedule of enrollment, interventions, and assessment. TECAS, transcutaneous electrical cranial-auricular acupoint stimulation; HAM-D, 17-item hamilton depression rating scale; HADRS, montgomery–åsberg depression rating scale; HAM-A, hamilton anxiety rating scale; PSQI, Pittsburgh sleep quality index; SF-36, short form 36 health survey.

The secondary outcomes include (a) response rate based on HAMD-17. The responder is defined as the rate of a ≥50% reduction from baseline HAMD-17 at week 8; (b) remission rate, which is defined as a score of 12 or fewer points on the MADRS or as 7 or fewer points on the HAMD-17 at the end of treatment; (c) changes from baseline in the MADRS at week 2, 4, 8, and 12; (d) changes from baseline in the HAMA at week 2, 4, 8, and 12; (e) changes from baseline in the PSQI at week 2, 4, 8, and 12; (f) changes from baseline in the SF-36 at week 2, 4, 8, and 12; (g) changes from baseline in the HAMD-17 at week 2, 4, 8, and 12. HAMD-17 is a validated questionnaire that needs to be assessed by clinicians (42). The range of HAMD-17 is 0–52 (higher scores indicate more severe condition). HAMA is a validated 14-item scale for the measurements of psychic anxiety and somatic anxiety (43). Each item of HAMA ranges from 0 to 4, with a total range of 0–56. PSQI consists of 19 items, and is a reliable self-reported questionnaire for the measurement of sleep dysfunction (higher scores indicate more severe condition) (44). SF-36 is made up of 36 items grouped in 8 dimensions (45). This scale is used to assess the overall health status of patients. Figure 3 illustrates the SPIRIT diagram of assessments at enrollment, allocation, and different time points.

#### Safety Assessment

All associated research staff will be trained in the recognition of and response to AE, serious adverse event (SAE), and suspected unexpected serious adverse reaction (SUSAR) according to the standard operating procedure approved by the Institutional Review Board of The University of Hong Kong/Hospital Authority Hong Kong West Cluster. All possible events will be independently evaluated by staff who do not assess treatment outcomes. The study team will follow up with participants during the whole study period and record all adverse events on a regular basis (baseline, 2, 4, 8, and 12 weeks). In general, TECAS treatment is relatively safe, but side effects such as mild dizziness, headache and local skin irritation from electrode placement are possible and will be of special concern. SAE for treatment discontinuation will be recorded including the occurrence time, severity, duration, measurement, management, and its outcome when such events occurred, and will be immediately reported to the principal investigator (Z-JZ) simultaneously. A safety report will be reviewed by the reviewers in the Expedited Panel through an expedited review process as stipulated. If the reviewers deem a safety report has any significant implication on the protection of patients’ safety, the report will be channeled for full review.

### Data Management

Data entry will be checked using a 5% double-entry procedure. Paper copies of measures are stored in locked filling cabinets. Anonymized measures data will be stored electronically on the National Population Health Data Center/Population Health Data Archive approved by re3data and FAIRsharing with password-protected secure, only principal investigator (Z-JZ) having access.

### Statistical Analysis

The proportion of participants with a reduction ≥50% in MADRS from baseline was 83.3% in the TECAS group and was 80.0% in the escitalopram group in our pilot study (unpublished data). Calculated by PROC POWER in SAS, in total, 470 participants will be needed to provide 90% power to detect a difference of −10% (the non-inferior margin) between groups in the proportion of participants with ≥50% in MADRS after 8 weeks of treatment at a 1-sided significance level of 5%, assuming a 20% loss to follow-up rate. The primary outcome will be evaluated based on the per-protocol (PP) population, defined as all randomized populations without major protocol violations. The primary outcome will be assessed using a 1-tailed test at a significance level of 0.025, while secondary outcomes using a 2-tailed test at a significant level of 0.05. For the proportion of participants with a reduction ≥50% in MADRS from baseline, the Cochran-Mantel-Haenszel test, stratified by site, will be used to test a hierarchical comparison between two groups. For the change from baseline in MADRS, the longitudinal data will be analyzed by fitting linear mixed-effects models using the baseline value as a covariate and treatment, visit, and treatment-by-visit interaction as fixed effects. The same approach will be used for the other continuous variables with longitudinal data, including HAMD-17, HAMA, PSQI, and SF-36. Missing data for the primary outcome will be imputed using the multiple imputation method under the missing at random assumption. For secondary outcomes, no imputation will be performed. To assess the robustness of the primary analyses, a sensitivity analysis will be performed without multiple imputation. All statistical analyses will be performed using SAS software, version 9.4 (SAS Institute).

## Discussion

This study uses a non-inferiority design to compare the effectiveness of TECAS and escitalopram for depression with large sample size. This design approach will help us to determine whether TECAS, a novel therapeutic method, is clinically equivalent to the current standard first-line antidepressants with fewer adverse events.

The response rate that will be determined by MADRS is chosen as the primary outcome. The well-established MADRS is one of the most widely used scales in psychopharmacological drug research (39). The scale is less influenced by adverse patient personality traits and more focused on the assessment of core depressive symptoms, which can respond well to changes in depression severity (46). Also, we will use the HAMD-17, a scale with a high degree of recognition, as a secondary outcome to assist in evaluating the severity of depression. Insomnia and anxiety accompanying depression, and quality of life will be assessed as well. This allows for a more comprehensive comparison of the differences between TECAS and escitalopram in terms of treatment effects. In addition to comparing the therapeutic efficacy at the treatment endpoint, a 4-week follow-up period is established. This is used to compare the difference between TECAS and escitalopram in terms of the durability of the treatment effect.

There are some limitations to this trial. Firstly, it is almost impossible to blind patients and therapists due to the huge operational variability between drug treatment and TECAS. However, this trial will be blinded to the assessor to minimize the potential for bias. Secondly, as TECAS is a novel fusional treatment, its application lacks widely used, or standardized, optimal parameters, such as frequency, amperage, and duration. We expect the results of this trial to demonstrate the potential of TECAS as an optional treatment for depression in the future, and further confirm the optimal parameters and optimize its treatment outcomes in subsequent applications and studies.

## Ethics Statement

The studies involving human participants were reviewed and approved by the Human Research Ethics Committees at The University of Hong Kong Shenzhen Hospital (Approval Number 2019044; approved 28 February 2019). The patients/participants have to provide their written informed consent to participate in this study.

## Author Contributions

Z-JZ and PR contributed to the conception of the study. SY and ZQ drafted the manuscript. ZQ developed the statistical analysis plan. All authors were involved in the design of the project, read, and approved the final manuscript.

## Conflict of Interest

The authors declare that the research was conducted in the absence of any commercial or financial relationships that could be construed as a potential conflict of interest.

## Publisher’s Note

All claims expressed in this article are solely those of the authors and do not necessarily represent those of their affiliated organizations, or those of the publisher, the editors and the reviewers. Any product that may be evaluated in this article, or claim that may be made by its manufacturer, is not guaranteed or endorsed by the publisher.

## Abbreviations

AE: adverse events
HAMA: hamilton anxiety rating scale
HAMD-17: 17-item hamilton depression rating scale
HKU-SZH: University of Hong Kong Shenzhen Hospital
ICD-10: international classification of diseases 10th edition
iVNS: implantable vagus nerve stimulation
MADRS: montgomery-åsberg depression rating scale
MCID: minimal clinically important difference
MDD: major depressive disorder
PSQI: Pittsburgh sleep quality index
SAE: serious adverse event
SF-36: short form 36 health survey
SNRI: serotonin and norepinephrine reuptake inhibitors
SPIRIT: standard protocol items: Recommendations for intervention trials
SSRI: selective serotonin reuptake inhibitor
SUSAR: suspected unexpected serious adverse reaction
TEAS: transcutaneous electrical acupoint stimulation
TECAS: transcutaneous electrical cranial-auricular acupoint stimulation
tVNS: transcutaneous vagus nerve stimulation
VNS: vagus nerve stimulation.
